# Life's Essential 8 and the Risk of Overall and Specific‐Site Cancer Types: A Large‐Scale Prospective Cohort Study

**DOI:** 10.1002/cam4.71518

**Published:** 2026-01-19

**Authors:** Li Deng, Xiao‐Meng Hao, Zhe Shu, Tong Liu, Qing‐Song Zhang, Hong‐Tao Wang, Han‐Ping Shi

**Affiliations:** ^1^ Department of Gastrointestinal Surgery, Department of Clinical Nutrition, Beijing Shijitan Hospital Capital Medical University Beijing China; ^2^ National Clinical Research Center for Geriatric Diseases, Xuanwu Hospital Capital Medical University Beijing China; ^3^ Key Laboratory of Cancer FSMP for State Market Regulation Beijing China; ^4^ Laboratory for Clinical Medicine Capital Medical University Beijing China; ^5^ The Third Department of Breast Cancer Tianjin Medical University Cancer Institute and Hospital Tianjin China; ^6^ Department of Medical Records Tianjin Medical University Cancer Institute and Hospital Tianjin China; ^7^ Department of General Surgery Kailuan General Hospital Tangshan China

**Keywords:** cancer, Life's Essential 8, prospective, protective

## Abstract

**Background:**

The Life's Essential 8 (LE8) framework, designed to prevent cardiovascular disease, is also linked to cancer risk, but the direct association between LE8 adherence and cancer incidence remains underexplored. Each LE8 component may affect cancer risk differently across organ systems, warranting further investigation into these relationships.

**Methods:**

This prospective cohort study utilized data from the Kailuan Study, comprising 94,239 participants from Tangshan, China. LE8 scores were calculated based on lifestyle and physiological measures, including diet, physical activity, and biomarkers such as blood glucose and cholesterol levels. Incident cancer cases were monitored through biennial health assessments and verified through multiple data sources until December 31, 2022. Cox proportional hazards models and competing risk models adjusted for confounders like age, sex, and lifestyle factors were used to analyze the impact of LE8 as well as each LE8 component on various cancer types.

**Results:**

During a median follow‐up of 14.99 years, 5124 incident cancer cases were identified. Compared to poor LE8 adherence, intermediate and ideal adherence groups showed a 13% (HR = 0.87; 95% CI: 0.80–0.96) and 26% (HR = 0.74; 95% CI: 0.65–0.85) decrease in overall cancer incidence, respectively. Improvements in LE8 scores were linked to reduced risks of specific cancers: lung (9%), breast (12%), kidney (18%), colorectal (7%), and endometrial (31%), with hazard ratios of 0.91, 0.88, 0.82, 0.93, and 0.69, respectively. Ideal blood pressure and non‐smoking were protective against cancer, while ideal body weight increased the risk. Non‐smoking reduced lung and kidney cancer risk, while ideal BMI had mixed effects.

**Conclusions:**

Our findings support the LE8 framework as an effective tool for cancer prevention, with higher adherence linked to lower cancer incidence across various types. The results advocate for the integration of LE8 into broader public health and clinical strategies.

**Trial Registration:**

Kailuan study, ChiCTR–TNRC–11001489. Registered August 24, 2011‐Retrospectively registered, http://www.chictr.org.cn/showprojen.aspx?proj=8050

**Abbreviations**
ALT: alanine aminotransferase
BMI: body mass index
CI: confidence intervals
CKD: chronic kidney disease
CPNI: cholesterol‐modified prognostic nutritional index
CRP: C‐reactive protein
CS: cause‐specific hazards
CVD: cardiovascular disease
DBP: diastolic blood pressure
FBG: fasting blood glucose
HBsAg: hepatitis B surface antigen
HDL‐C: High‐density lipoprotein cholesterol
HR: hazard ratios
IQR: interquartile range
LE8: Life's Essential 8
SBP: systolic blood pressure
SD: sub‐distribution hazards
TC: total cholesterol
WC: waist circumference

## 1 Background

The “Life's Essential 8” (LE8) framework, developed by the American Heart Association (AHA), represents a significant advancement from its predecessor, “Life's Simple 7,” with the addition of the sleep score. This updated model includes eight critical domains: diet, physical activity, nicotine exposure, sleep duration, body weight, blood lipids, blood glucose, and blood pressure [1]. Collectively, these domains form the cornerstone of strategies for the prevention and management of cardiovascular disease (CVD). Adherence to the LE8 guidelines is strongly associated with a reduced risk of CVD, emphasizing the importance of a holistic approach to health promotion and disease prevention [2]. Moreover, recent studies indicate that even modest improvements in these lifestyle factors can significantly reduce the risk of conditions like non‐alcoholic fatty liver disease [3] and stroke [4], suggesting the potential for widespread public health benefits through the adoption of the LE8 framework.

The relevance of the LE8 extends beyond cardiovascular health, also significantly impacting cancer outcomes. Diets high in processed and red meats have been associated with an increased risk of colorectal and other forms of cancer [5, 6, 7], while physical inactivity and obesity are recognized as independent risk factors for several types of cancer, such as breast and colorectal cancers [8, 9, 10]. Nicotine exposure, particularly through smoking, is a significant carcinogen, linked to a variety of cancers such as lung, throat, and bladder cancers [11, 12, 13]. Additionally, emerging research suggests that disruptions in sleep patterns may also contribute to cancer risk, potentially through circadian rhythm disturbances [14, 15, 16]. The integration of these lifestyle factors into cancer prevention strategies is increasingly recognized as a critical component of comprehensive cancer control plans.

While research on LE8 and cancer incidence remains limited [17], no studies have specifically examined the association between LE8 and site‐specific cancers, nor the relationship between each LE8 component with overall and site‐specific cancer risk. To address this gap, we utilize data from the Kailuan Prospective Study to comprehensively investigate the impact of LE8 adherence and its individual components on cancer risk. This study aims to provide novel insights into the role of LE8 in cancer prevention and management.

## 2 Methods

### 2.1 Study Populations

The Kailuan Study, as previously described [18, 19], is a longitudinally designed cohort investigation located in the Kailuan community, Tangshan, China. It targets a demographic that includes both currently employed and retired personnel from the Kailuan Company. The initial cohort included 101,510 participants (comprising 81,110 males and 20,400 females, ranging in age from 18 to 98 years) during the first health assessment phase, which spanned from July 2006 to October 2007. Since then, the study has conducted biennial health assessments for all participants. Researchers performed baseline assessments and biennial follow‐ups through direct standardized questionnaire interviews, physical assessments, clinical evaluations, and laboratory analyses. We excluded participants from the current study for several reasons: (1) a baseline history of cancer (*n* = 377); (2) incomplete data for LE8 components (*n* = 4502); (3) absence of information on other possible confounding factors (*n* = 2392). Consequently, the final analysis comprised 94,239 participants (as shown in Figure S1). The Ethics Committees at Kailuan General Hospital and Beijing Shijitan Hospital approved the study. All participants or their designated family members provided informed consent.

### 2.2 Measurement and Quantification of LE8 Score

LE8 scores were assessed utilizing the algorithm developed by the AHA for LE8 [20]. This evaluation assessed eight key metrics, which included both health behaviors (such as diet, physical activity, smoking, and sleep) and health factors (Body Mass Index (BMI), non‐high‐density lipoprotein cholesterol (HDL‐C), blood glucose, and blood pressure). Each metric was scored on a scale from 0 to 100, with the aim of measuring the continuum of health. The composite LE8 score was derived from the arithmetic mean of the scores across all eight metrics. Participants were classified based on their overall scores: 80–100 points for ideal, 50–79 for intermediate, and 0–49 for poor levels. The specifics of the measurements, definitions, and scoring criteria were detailed in Table S1. Data regarding dietary habits, physical activity levels, smoking status, and sleep duration were gathered using a uniform questionnaire by interviewers who had undergone specific training and certification. Body weight and height were recorded with participants in light clothing and barefoot, enabling BMI calculation as weight (kg) divided by height (m^2^). The World Health Organization guidelines were used for BMI scoring: 100 for a BMI of 18.5–22.9, 75 for 23.0–24.9, 50 for 25.0–29.9, 25 for 30.0–34.9, and 0 for 35.0 or higher. Blood pressure was measured twice after the participant rested for 5 min. A calibrated mercury sphygmomanometer was used for both readings, and the average of the two measurements was recorded. Participants fasted for at least 8 h before blood samples were collected for serum glucose and lipid levels. Non HDL‐C levels were calculated by subtracting HDL‐C from total cholesterol (TC).

### 2.3 Outcome Ascertainment

Cancer incidents were detected through a combination of methods until December 31, 2022: biennial participant self‐reports of symptoms or previous diagnoses; review of medical records from the Tangshan Medical Insurance System, Kailuan Social Security System, and Provincial Vital Statistics; and analysis of discharge records from 11 associated hospitals. Specialists confirmed all cancer diagnoses using either distinct clinical indicators or histopathological evidence obtained at the treating facilities. Cancer diagnoses were categorized based on the International Classification of Diseases, Tenth Revision (ICD‐10), as detailed in Table S2. Additionally, fatalities were ascertained via the Kailuan Social Security System.

### 2.4 Assessment of Confounders

Sociodemographic and lifestyle characteristics were obtained from standardized questionnaires administered at baseline. Variables included age, sex, household income (categorized as ≥ 1000 ¥ per month, cut‐off based on 2006 data), and educational attainment (high school level or above). Lifestyle exposures encompassed daily alcohol intake of ≥ 100 mL and prolonged sedentary behavior, defined as sitting for at least 8 h per day across occupational, household, or leisure contexts. Clinical parameters were also evaluated. Hypertension was classified according to the standard threshold of SBP ≥ 140 mmHg, DBP ≥ 90 mmHg, a physician's diagnosis, or current antihypertensive use. Diabetes mellitus was identified if fasting plasma glucose was ≥ 7.0 mmol/L, or if participants reported a history of diabetes or antidiabetic treatment. Laboratory testing included C‐reactive protein (CRP), alanine aminotransferase (ALT), and hepatitis B surface antigen (HBsAg), which were analyzed centrally using automated biochemical analyzers (Hitachi 747). Hepatobiliary conditions such as cirrhosis, steatosis, gallstones, and gallbladder polyps were ascertained by abdominal ultrasonography (Philips HD‐15) or verified against medical records from Tangshan healthcare facilities.

### 2.5 Statistical Analysis

We analyzed continuous variables using means with standard deviations when distributions were normal, and medians with interquartile ranges when skewed. We tested group differences with analysis of variance (ANOVA) for normal data and with the Kruskal–Wallis test for skewed data. We expressed categorical variables as counts with percentages and compared them using the Chi‐square test. We computed person‐years from the baseline examination (2006–2007) until the earliest of cancer diagnosis, death, or December 31, 2021. To evaluate cancer risk, we built Cox proportional hazards models and calculated hazard ratios (HRs) with 95% confidence intervals (CIs). For site‐specific cancers, we adjusted models for relevant covariates and stratified analyses by sex where appropriate. We applied restricted cubic splines (RCS) to explore nonlinear associations between LE8 and cancer outcomes, log‐transforming HRs to stabilize variance and aid interpretation. To account for competing risks such as non‐cancer deaths, we fitted both cause‐specific hazard models and Fine–Gray subdistribution hazard models.

To minimize reverse causation, we excluded individuals who developed cancer within the first year in sensitivity analyses. It is noted that LE8 does not consider alcohol consumption, which significantly impacts cancer risk, and links to chronic inflammation, a key factor in cancer development. Subgroup analyses were based on alcohol consumption and chronic inflammation (CRP > 3 mg/L). All analyses were performed with a significance level of < 0.05 using SAS software, version 9.4.

## 3 Results

This study enrolled 94,239 participants, consisting of 75,327 (79.93%) men and 18,912 (20.07%) women, with an average age of 51.48 ± 12.47 years (ranging from 18 to 98 years). Participants were categorized into three groups based on the LE8 level: poor (LE8 < 50; *n* = 8641), intermediate (LE8: 50–79; *n* = 76,055), and ideal (LE8 ≥ 80; *n* = 9543). Baseline demographic and clinical characteristics, stratified by LE8 level, are presented in Table 1. Statistically significant intergroup differences were observed in various baseline characteristics, including age, BMI, WC, SBP, DBP, FBG, TC, HDL‐C, TG, CRP, ALT, sex distribution, per capita household income, educational background, physical activity levels, smoking and alcohol consumption habits, salt and tea consumption, intake of a high‐fat diet, and the prevalence of diabetes, hypertension, fatty liver, gallbladder polyps, and HBsAg seropositivity (all *p*‐values < 0.05).

**TABLE 1:** Baseline characteristics of the participants stratified by the cutoff of LE8.

Variables	Overall	LE8 scores by cutoff			
		Poor (< 50)	Intermediate (50–79)	Ideal (≥ 80)	*p*
*n* (%)	94,239	8641	76,055	9543	
Men (%)	75,327 (79.93)	8253 (95.51)	62,201 (81.78)	4873 (51.06)	< 0.001
Age (year)	51.48 ± 12.47	52.75 ± 10.47	52.40 ± 12.35	45.83 ± 13.57	< 0.001
BMI (kg/m^2^)	25.46 ± 3.34	27.31 ± 3.44	25.20 ± 3.36	21.93 ± 2.32	< 0.001
WC (cm)	86.98 ± 9.66	92.26 ± 9.17	87.29 ± 9.59	79.38 ± 9.45	< 0.001
SBP (mmHg)	131.06 ± 21.07	147.24 ± 20.94	131.72 ± 20.11	111.28 ± 11.46	< 0.001
DBP (mmHg)	83.63 ± 11.62	92.08 ± 12.40	83.88 ± 11.24	73.77 ± 8.00	< 0.001
FBG (mmol/L)	5.49 ± 1.69	6.73 ± 2.62	5.43 ± 1.57	4.82 ± 0.58	< 0.001
TC (mmol/L)	4.95 ± 1.16	5.78 ± 1.29	4.93 ± 1.13	4.38 ± 0.77	< 0.001
HDL‐C (μmol/L)	1.55 ± 0.39	1.52 ± 0.39	1.55 ± 0.40	1.57 ± 0.38	< 0.001
TG (mmHg)	1.29 (0.90, 1.93)	1.74 (1.19, 2.67)	1.29 (0.92, 1.94)	0.91 (0.66, 1.26)	< 0.001
CRP (mg/L)	0.81 (0.30, 2.05)	1.10 (0.48, 2.50)	0.80 (0.30, 2.07)	0.50 (0.20, 1.50)	< 0.001
ALT (u/L)	17.9 (12.8, 24.9)	20.0 (13.0, 28.0)	18.0 (13.0, 25.0)	15.0 (11.0, 21.0)	< 0.001
Per capita income (> 1000 ¥ in 2006)	6257 (6.64)	615 (7.12)	4794 (6.30)	848 (8.89)	< 0.001
Education (High school or above, %)	18,668 (19.81)	1403 (16.24)	14,062 (18.49)	3203 (33.56)	< 0.001
Physical exercise (%)					< 0.001
Never	8273 (8.78)	2726 (31.55)	5491 (7.22)	56 (0.59)	
Occasionally	71,158 (75.51)	5155 (59.66)	58,708 (77.19)	7295 (76.44)	
Regularly	14,808 (15.71)	760 (8.80)	11,856 (15.59)	2192 (22.97)	
Smoking status (%)					< 0.001
Never	56,240 (59.68)	938 (10.86)	46,099 (60.61)	9203 (96.44)	
Former smoker	5401 (5.73)	589 (6.82)	4642 (6.10)	170 (1.78)	
Current smoker, < 1 cigarette/day	3365 (3.57)	420 (4.86)	2893 (3.80)	52 (0.54)	
Current smoker, ≥ 1 cigarette/day	29,233 (31.02)	6694 (77.47)	22,421 (29.48)	118 (1.24)	
Drinking status (%)					< 0.001
Never	55,491 (58.88)	2344 (27.13)	45,582 (59.93)	7565 (79.27)	
Former drinker	3666 (3.89)	585 (6.77)	2939 (3.86)	142 (1.49)	
Current drinker, < 100 mL/day	18,149 (19.26)	2156 (24.95)	14,551 (19.13)	1442 (15.11)	
Current drinker, ≥ 100 mL/day	16,933 (17.97)	3556 (41.15)	12,983 (17.07)	394 (4.13)	
Dietary salt consumption					< 0.001
< 6 g/day	8715 (9.25)	710 (8.22)	6722 (8.84)	1283 (13.44)	
6–12 g/day	75,303 (79.90)	5623 (65.07)	61,813 (81.27)	7867 (82.44)	
> 12 g/day	10,221 (10.85)	2308 (26.71)	7520 (9.89)	393 (4.12)	
Tea consumption					
< 1 time/month	69,146 (73.37)	6509 (75.33)	57,433 (75.52)	5204 (54.53)	
1–3 times/month	10,413 (11.05)	1102 (12.75)	8016 (10.54)	1295 (13.57)	
1–3 times/week	8886 (9.43)	1020 (11.80)	7030 (9.24)	836 (8.76)	
≥ four times/week	5794 (6.15)	10 (0.12)	3576 (4.70)	2208 (23.13)	
High fat diet					< 0.001
< 1 time/week	7983 (8.47)	623 (7.21)	6135 (8.07)	1225 (12.84)	
1–3 times/week	77,497 (82.23)	6043 (69.93)	63,464 (83.44)	7990 (83.73)	
> 3 times/week	8759 (9.29)	1975 (22.86)	6456 (8.49)	328 (3.44)	
Diabetes mellitus (%)	8457 (8.97)	2360 (27.31)	6042 (7.94)	55 (0.58)	< 0.001
Hypertension (%)	34,856 (36.99)	6059 (70.12)	28,467 (37.43)	330 (3.46)	< 0.001
Fatty liver (%)	24,297 (25.78)	2864 (33.14)	20,751 (27.28)	682 (7.15)	< 0.001
Gallbladder polyp (%)	901 (0.96)	775 (1.16)	90 (1.15)	36 (1.03)	< 0.001
HBsAg Seropositive (%)	769 (0.82)	50 (0.58)	616 (0.81)	103 (1.08)	0.001

Abbreviations: ALT, alanine aminotransferase; BMI, body mass index; CRP, C‐reactive protein; DBP, diastolic blood pressure; FBG, fasting blood glucose; SBP, systolic blood pressure; TC, total cholesterol; TG, triglyceride; WC, waist circumference.

**FIGURE 1:**
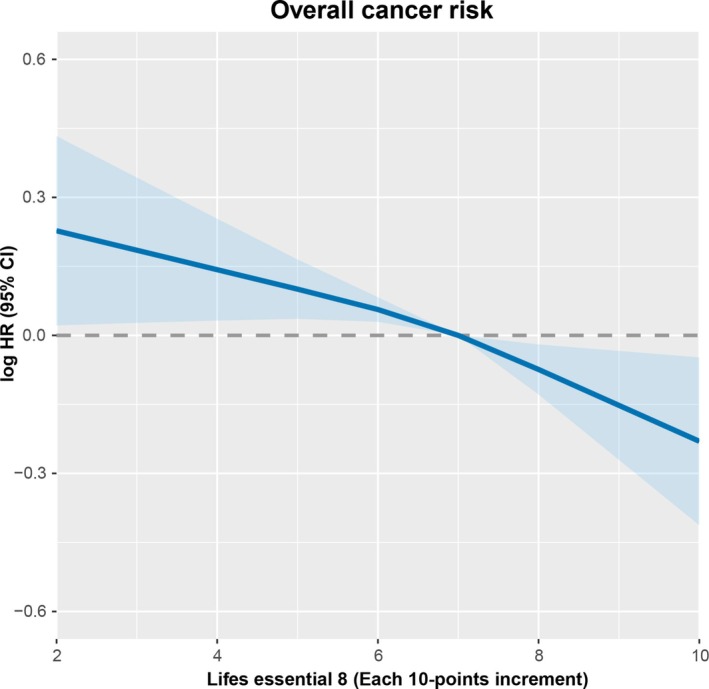
The adjusted dose–response relationship between LE8 and overall cancer risk by using RCS.

During a median follow‐up of 14.99 years (IQR: 14.61–15.19), a total of 5124 incident cancer cases were identified. Figure 1 illustrates the dose–response relationship between LE8 and overall cancer risk, showing a linear and negative association, where higher LE8 scores are associated with a decreased cancer risk. Table 2 presents the associations between continuous increments of LE8 (per 10‐point increase), categorizations by cutoffs, and quartiles with overall cancer risk. After adjusting for confounders such as age, sex, and alcohol consumption, each 10‐point increase in LE8 was associated with a 6% decreased risk for overall cancer incidence (HR = 0.94; 95% CI: 0.91–0.96). Compared to the poor group, the intermediate and ideal groups showed a 13% (HR = 0.87; 95% CI: 0.80–0.96) and 26% (HR = 0.74; 95% CI: 0.65–0.85) reduction in cancer incidence risk, respectively. Furthermore, the highest quartile group was associated with a 17% reduction in cancer incidence risk (HR = 0.83; 95% CI: 0.76–0.90) compared to the lowest quartile.

**TABLE 2:** Adjusted HRs and 95% CIs for risk of overall cancer across Life's Essential 8 scores quartiles and clinical cutoffs in Kailuan Study.

	Cases/participants	Cox regression		CS models		SD models	
		HRs (95% CI)	*p*	HRs (95% CI)	*p*	HRs (95% CI)	*p*
Each 10‐points increment	5124/89,115	**0.94 (0.91, 0.96)**	**< 0.001**	**0.97 (0.94, 0.99)**	**0.017**	0.98 (0.95, 1.01)	0.071
LE8 scores by cutoff							
Poor (< 50)	552/8641	Ref.		Ref.		Ref.	
Intermediate (50–79)	4347/76,055	**0.87 (0.80, 0.96)**	**0.003**	0.96 (0.88, 1.06)	0.133	0.99 (0.92,1.10)	0.217
Ideal (≥ 80)	385/9543	**0.74 (0.65, 0.85)**	**< 0.001**	**0.88 (0.75, 0.98)**	**0.007**	**0.89 (0.76, 0.99)**	**0.012**
*p* for trend		**< 0.001**		0.160		0.247	
LE8 scores by quartiles							
Q1 (< 58.34)	1443/23,749	Ref.		Ref.		Ref.	
Q2 (58.34–66.67)	1501/25,267	0.95 (0.88, 1.02)	0.181	0.98 (0.91, 1.06)	0.231	0.99 (0.92, 1.08)	0.227
Q3 (66.68–74.17)	1344/23,172	0.96 (0.88, 1.04)	0.277	0.99 (0.92, 1.10)	0.291	1.00 (0.93, 1.12)	0.301
Q4 (74.17)	996/22,051	**0.83 (0.76, 0.90)**	**< 0.001**	**0.87 (0.80, 0.95)**	**< 0.001**	**0.88 (0.81, 0.97)**	**0.001**
*p* for trend		**< 0.001**		**< 0.001**		**0.011**	

*Note:* Adjusted models include age, gender, CRP, drinking status, marital status, family income, and sedentary lifestyles. Results presented with bold valued were statistically significant with all *p* value < 0.05.

Abbreviations: CS, cause‐specific hazards; SD, sub‐distribution hazards.

Table 3 displays the relationship between the categorical and continuous forms of the LE8 score and the risk of specific‐site cancers. For every 10‐point increase in the continuous LE8 score, there was a reduction in the risk of lung (9%), breast (12%), kidney (18%), colorectal (7%), and endometrial cancer (31%), with corresponding HRs (95% CIs) of 0.91 (0.86–0.96), 0.88 (0.79–0.99), 0.82 (0.72–0.94), 0.93 (0.86–0.99), and 0.69 (0.52–0.92), respectively. In the categorical analysis of LE8, compared to the “poor” group, the “intermediate” group showed a 20% and 61% decrease in the risk of lung (HR = 0.80, 95% CI: 0.68–0.94) and breast cancer (HR = 0.39, 95% CI: 0.24–0.63), respectively. The “ideal” group exhibited reductions in the incidence of lung (26%), breast (65%), kidney (56%), and colorectal cancer (52%), with HRs of 0.74 (95% CI: 0.57–0.95), 0.35 (95% CI: 0.20–0.61), 0.44 (95% CI: 0.20–0.96), and 0.48 (95% CI: 0.31–0.73), respectively. Although the continuous form of LE8 was associated with a reduced risk of endometrial cancer, the case number was too small for categorical analysis, so we did not proceed with further classification.

**TABLE 3:** Adjusted HRs and 95% CIs for risk of specific‐site cancer across the clinical cutoffs of Life's Essential 8 scores in Kailuan Study.

Cancer types		Each 10‐points increment	LE8 scores by cutoff			*p* for trend
			Poor (< 50)	Intermediate (50–79)	Ideal (≥ 80)	
Colorectal cancer	No. of cases	730	90	610	30	
	Cox models	**0.93 (0.86, 0.99)**	Ref.	0.84 (0.67, 1.06)	**0.48 (0.31, 0.73)**	**0.003**
	CS models	0.93 (0.86, 1.00)	Ref.	0.84 (0.67, 1.06)	**0.48 (0.31, 0.73)**	**0.003**
	SD models	0.94 (0.88, 1.01)	Ref.	0.88 (0.70, 1.10)	**0.49 (0.32, 0.75)**	**0.004**
Lung cancer	No. of cases	1507	176	1226	105	
	Cox models	**0.91 (0.86, 0.96)**	Ref.	**0.80 (0.68, 0.94)**	**0.74 (0.57, 0.95)**	**0.018**
	CS models	**0.91 (0.86, 0.96)**	Ref.	**0.80 (0.68, 0.94)**	**0.74 (0.57, 0.95)**	**0.017**
	SD models	**0.92 (0.88, 0.97)**	Ref.	**0.83 (0.70, 0.98)**	**0.77 (0.59, 0.98)**	0.057
Breast cancer^a^	No. of cases	323	20	243	60	
	Cox models	**0.88 (0.79, 0.99)**	Ref.	**0.39 (0.24, 0.63)**	**0.35 (0.20, 0.61)**	**< 0.001**
	CS models	**0.88 (0.77, 0.99)**	Ref.	**0.39 (0.24, 0.63)**	**0.35 (0.20, 0.62)**	**< 0.001**
	SD models	0.88 (0.77, 1.01)	Ref.	**0.41 (0.25, 0.66)**	**0.36 (0.21, 0.64)**	**< 0.001**
Kidney cancer	No. of cases	218	27	182	9	
	Cox models	**0.82 (0.72, 0.94)**	Ref.	NA	NA	0.121
	CS models	**0.82 (0.72, 0.95)**	Ref.	NA	NA	0.120
	SD models	**0.83 (0.74, 0.95)**	Ref.	NA	NA	0.116
Endometrial cancer^a^	No. of cases	58	1	51	6	
	Cox models	**0.69 (0.52, 0.92)**	NA	NA	NA	NA
	CS models	**0.74 (0.58, 0.95)**	NA	NA	NA	NA
	SD models	**0.70 (0.54, 0.89)**	NA	NA	NA	NA
Bladder cancer	No. of cases	189	21	159	9	
	Cox models	1.00 (0.86, 1.15)	Ref.	NA	NA	0.397
Prostatic cancer^a^	No. of cases	156	141	7	8	
	Cox models	0.96 (0.82, 1.13)	Ref.	NA	NA	0.058
Esophageal cancer	No. of cases	200	21	174	5	
	Cox models	0.92 (0.80, 1.06)	Ref.	NA	NA	0.107
Stomach cancer	No. of cases	403	44	338	21	
	Cox models	0.92 (0.84, 1.02)	Ref.	0.85 (0.61, 1.18)	0.60 (0.35, 1.04)	0.184
Head and neck cancer	No. of cases	459	44	366	49	
	Cox models	0.98 (0.89, 1.07)	Ref.	0.87 (0.63, 1.20)	0.89 (0.57, 1.38)	0.684
Pancreatic cancer	No. of cases	141	17	117	7	
	Cox models	0.92 (0.78, 1.08)	Ref.	NA	NA	0.348
Liver cancer^b^	No. of cases	458	54	371	33	
	Cox models	0.91 (0.83, 1.00)	Ref.	0.76 (0.57, 1.03)	0.73 (0.46, 1.16)	0.191

*Note:* Adjusted models include age, gender, CRP, drinking status, marital status, family income, and sedentary lifestyles. Results presented with bold valued were statistically significant with all *p* value < 0.05.

Abbreviations: CS, cause‐specific hazards; SD, sub‐distribution hazards.

^a^ Conducted only in men or women.

^b^ Further adjusted for ALT, HBV infection, liver cirrhosis, and fatty liver disease.

Over a median follow‐up period of 14.99 years, 11,122 deaths from non‐cancer causes occurred before the diagnosis of cancer; these were treated as competing risks in our analysis to better observe cancer incidence. To address this, we applied competing risk models. The findings suggest that LE8 is associated with a reduced risk of developing overall cancer, lung cancer, breast cancer, renal cancer, and colorectal cancer, as demonstrated in both the CS and SD models (Tables 2 and 3).

**FIGURE 2:**
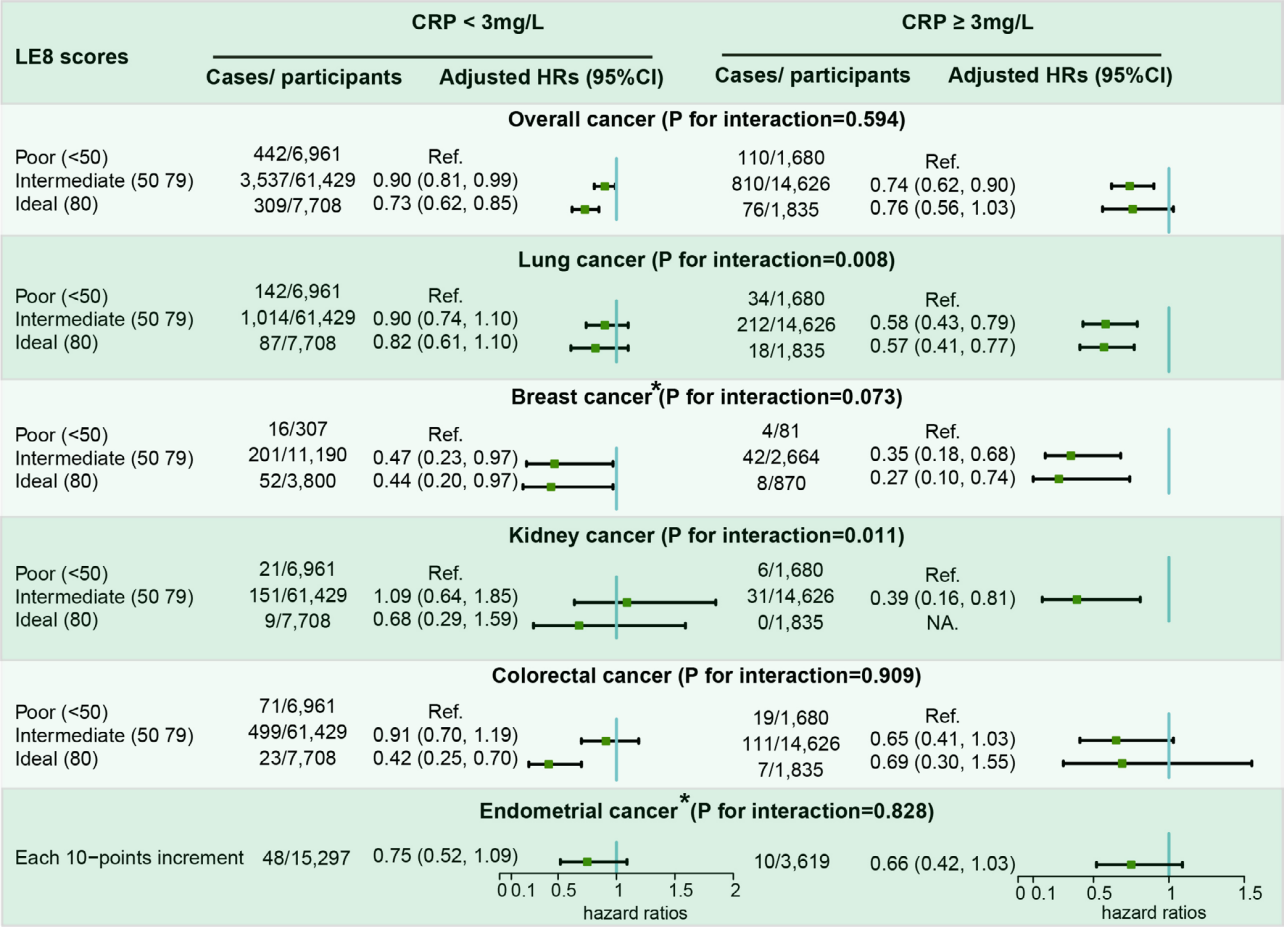
Adjusted HRs and 95% CIs for risk of specific‐site cancer across the clinical cutoffs of Life's Essential 8 scores stratified by CRP levels. Adjusted models include age, gender, drinking status, marital status, family income, and sedentary lifestyles. *: conducted only in women.

**FIGURE 3:**
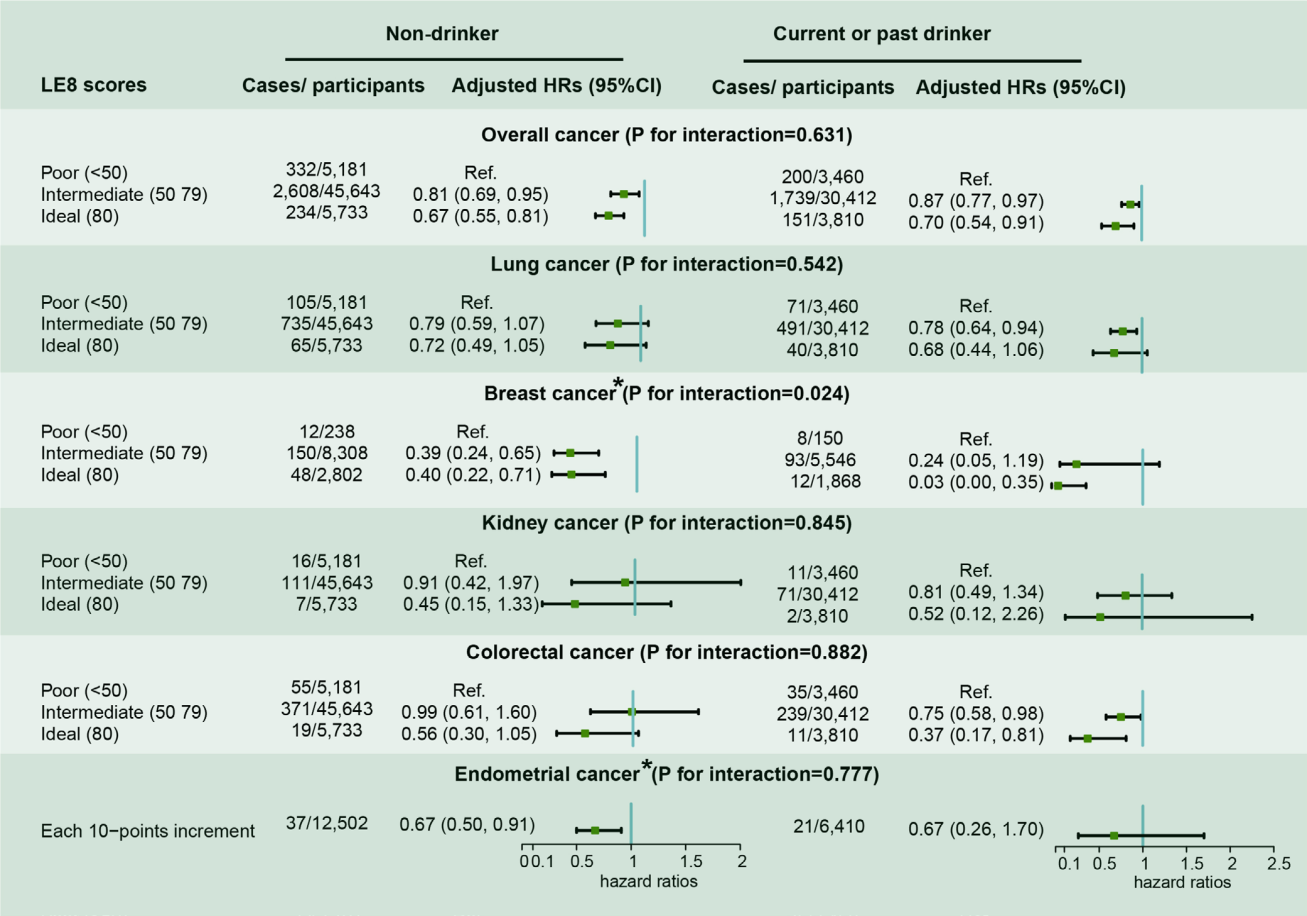
Adjusted HRs and 95% CIs for risk of specific‐site cancer across the clinical cutoffs of Life's Essential 8 scores stratified by drinking status. Adjusted models include age, gender, drinking status, marital status, family income, and sedentary lifestyles. *: conducted only in women.

In the subgroup analyses, we further explored how CRP levels and alcohol consumption influenced the associations of LE8 with cancer risks. For individuals with low CRP levels (< 3 mg/L), LE8 was associated with decreased risks of overall, breast, and colorectal cancers. In individuals with high CRP levels (≥ 3 mg/L), LE8 was linked to lower risks of overall, lung, breast, and kidney cancers. Notably, there was a significant interaction between CRP levels and LE8 in affecting lung and kidney cancer risks, as illustrated in Figure 2. When categorizing participants by drinking status, LE8 showed varied associations with cancer risks: among non‐smokers, it was associated with decreased risks of overall, breast, and endometrial cancers, while among smokers, it was linked to reductions in the risks of overall, lung, breast, and colorectal cancers. Importantly, drinking status significantly modified the association between LE8 and breast cancer risk, as depicted in Figure 3.

Table 4 shows the relationship between each component of LE8 and cancer risk. After adjusting for confounding factors, we observed that ideal blood pressure and non‐smoking were associated with a reduced risk of overall cancer incidence. However, an ideal body weight was linked to an increased risk of overall cancer incidence. Specifically, non‐smoking was associated with a reduced risk of lung and kidney cancer. Ideal BMI was associated with an increased risk of lung cancer but a reduced risk of kidney, colorectal, and endometrial cancers. Ideal NON‐HDL cholesterol levels were associated with a reduced risk of breast cancer, while ideal blood pressure was associated with a reduced risk of kidney cancer. Lastly, ideal blood glucose levels were associated with a lower risk of colorectal and endometrial cancers.

**TABLE 4:** Adjusted HRs and 95% CIs of each LE8 component for risk of overall and specific‐site cancer in Kailuan Study.

	Overall cancer	Lung cancer	Breast cancer	Kidney cancer	Colorectal cancer	Endometrial cancer
Ideal blood pressure	**0.98 (0.97, 0.99)**	0.99 (0.96, 1.04)	0.99 (0.95, 1.05)	**0.96 (0.94, 0.98)**	0.98 (0.97, 1.01)	0.98 (0.96, 1.02)
Ideal blood glucose	1.01 (0.98, 1.05)	0.99 (0.97, 1.03)	1.03 (0.99, 1.08)	1.02 (0.98, 1.08)	**1.04 (1.02, 1.07)**	**1.03 (1.01, 1.07)**
Ideal BMI	**1.02 (1.01, 1.04)**	**1.06 (1.03, 1.10)**	0.97 (0.94, 1.02)	**0.93 (0.91, 0.96)**	**0.96 (0.94, 0.99)**	**0.96 (0.95, 0.99)**
Ideal Non‐HDL cholesterol	1.00 (0.98, 1.03)	0.98 (0.96, 1.01)	**0.96 (0.94, 0.99)**	1.00 (0.97, 1.04)	0.98 (0.94, 1.04)	0.98 (0.95, 1.03)
Regular physical activity	1.00 (0.97, 1.04)	0.98 (0.96, 1.01)	0.98 (0.95, 1.02)	1.01 (0.99, 1.04)	1.00 (0.98, 1.03)	1.01 (0.98, 1.05)
Non‐smoking status	**0.97 (0.95, 0.99)**	**0.95 (0.93, 0.97)**	0.97 (0.95, 1.01)	**0.94 (0.92, 0.97)**	0.99 (0.97, 1.03)	0.99 (0.96, 1.03)
Ideal diet	0.99 (0.96, 1.03)	0.98 (0.96, 1.01)	1.01 (0.98, 1.05)	1.01 (0.97, 1.07)	1.02 (0.98, 1.07)	1.00 (0.98, 1.03)
Ideal sleep	**1.00 (0.98, 1.03)**	**0.98 (0.96, 1.01)**	**1.00 (0.97, 1.04)**	**0.97 (0.94, 1.02)**	**1.01 (0.99, 1.05)**	**1.01 (0.98, 1.05)**

*Note:* Adjusted models include age, gender, CRP, drinking status, marital status, family income, sedentary lifestyles, and other components of LE8. Bold values indicate statistically significant.

In the sensitivity analysis, to mitigate the potential impact of preclinical disease on the results, we removed participants who developed any cancer‐related condition within the first year of follow‐up. LE8 continued to exhibit statistically significant protective effects against the development of overall cancer, lung cancer, breast cancer, renal cancer, and colorectal cancer (Table S3).

## 4 Discussion

In this large‐scale prospective cohort study, we observe that the LE8 score is strongly associated with the risk of cancer. Specifically, higher LE8 scores are associated with reduced risks for overall, lung, breast, colorectal, kidney, and endometrial cancers. Further analyses reveal that each component of LE8 has a varied impact on tumor development across different sites. This variation highlights the distinct risk factors for different types of tumors and underscores the importance of a comprehensive evaluation using the LE8 framework.

Our study suggests that adhering to LE8 significantly reduces the risk of developing several types of cancers, including lung, breast, colorectal, kidney, and endometrial cancer. This comprehensive health metric, which emphasizes optimal lifestyle and physiological measures, appears to have a broad protective effect against various types of cancer. In one of the previous studies, Jiang J et al. demonstrated a significant association between LE8 and the risk of death and cancer, particularly among younger adults. However, this study did not further explore the differential impact of LE8 on the incidence of specific cancer types or the individual effects of each LE8 component on cancer risk. Fan et al. [21] demonstrated a significant association between LE8 and reduced mortality in US cancer survivors, which suggests that the principles of LE8 may also contribute to lower cancer risk. Additionally, Kobo et al. [22] and Abramov et al. [23] provide insights into how cardiovascular health metrics, which are integral to LE8, differ among individuals with and without cancer and how these metrics are associated with mortality. Ogunmoroti and Osibogun [24] further discuss the potential of achieving optimal cardiovascular health as a preventative measure against cancer, underscoring the relevance of LE8 components in cancer prevention strategies.

While research on the relationship between LE8 and cancer incidence is limited, there has been extensive investigation into the association of LE8 with various chronic diseases. Specifically, the research by Wang et al. [3] and He et al. [25] underscores the significant impact of LE8 scores on the incidence of non‐alcoholic fatty liver disease in diverse populations. Additionally, Li et al. [2] highlighted the protective effects of high LE8 scores against cardiovascular diseases, even in individuals with genetic predispositions to these conditions. This suggests that LE8's health metrics are robust across different risk profiles. Furthermore, Zhang et al. [26] observed that better LE8 scores correlated with markers of reduced biological aging, providing evidence that this metric not only affects disease‐specific risks but also general health longevity. Moreover, Wu et al. [4] demonstrated that LE8 adherence significantly lowered stroke risk in a large‐scale community‐based study, reinforcing the cardiovascular benefits of LE8. Also, Ren et al. [27] explored the association between LE8 and chronic kidney disease (CKD) prevalence among US adults, utilizing data from 2007 to 2018. Their findings indicate that higher adherence to LE8 is significantly linked to lower CKD prevalence, underscoring the utility of LE8 metrics beyond cardiovascular health and highlighting its importance in renal health.

Our study reveals that LE8 significantly protects against lung and kidney cancer risks in individuals with elevated inflammation levels (CRP > 3 mg/L), highlighting a key interaction between LE8 and inflammation markers. Chronic inflammation is known to facilitate carcinogenesis by damaging DNA, promoting cell proliferation, and enhancing survival of malignant cells [28, 29]. LE8 likely modulates these inflammatory responses, potentially interfering with critical cytokines or pathways that accelerate cancer development in inflamed tissues [30]. Complementing this, Liu et al.'s prospective cohort study provides further evidence by demonstrating that distinct CRP trajectories are associated with varying risks for all types of cancer. Their findings suggest that individuals with consistently high or increasing CRP levels face a higher risk of developing cancer, reinforcing the importance of inflammation as a central factor in cancer risk and progression [31].

This research shows that maintaining an ideal BMI increases the risk of lung cancer but decreases the risk for kidney, colorectal, and endometrial cancers, illustrating the complex relationship between BMI and cancer risks. The “obesity paradox”—where obesity may offer survival benefits in certain cancers like lung cancer—provides context to these findings. This paradox is noted in lung cancer studies where obese patients exhibit better survival, likely due to greater metabolic reserves during treatment [32]. Nitsche et al. confirm this by showing that ideal BMI increases lung cancer risk but decreases it in other cancers [33]. Additionally, Vedire, Kalvapudi, and Yendamuri's review highlights that while obesity generally raises cancer risks, its specific effects on lung cancer involve complex biological interactions that could modify both risk and prognosis [34].

Our study indicates that optimal levels of non‐HDL‐C significantly reduce the risk of breast cancer. This finding is consistent with emerging research that highlights the integral role of lipid metabolism in breast cancer development and progression. For instance, the Cholesterol‐modified Prognostic Nutritional Index (CPNI) has been identified as an effective tool for assessing nutritional status and predicting survival in breast cancer patients. Shi et al. demonstrate that CPNI provides a more refined nutritional evaluation, which is crucial for predicting the prognosis of breast cancer patients, underscoring the connection between cholesterol levels and breast cancer outcomes [35]. Complementing this, the comprehensive review by Zipinotti Dos Santos et al. further elucidates how lipid metabolism contributes to breast cancer by facilitating tumorigenesis and aiding immune escape [36]. Their findings suggest that lipid metabolites may promote oncogenic pathways and enhance the tumor microenvironment's capacity to evade immune surveillance, thus playing a dual role in the progression and resilience of breast cancer.

Several underlying mechanisms could elucidate the inverse relationship between LE8 and subsequent cancer risk. First, reducing inflammation is a key mechanism where healthy lifestyle choices decrease the mutation rate and progression of precancerous cells, thus lowering cancer risk [37, 38]. Second, hormonal regulation plays a crucial role, particularly through weight management and dietary control, which help regulate hormone levels like estrogen and insulin that are linked to various cancers [39, 40]. Third, enhanced immune surveillance is facilitated by regular physical activity, allowing the body's immune system to better detect and eliminate cancer cells [41]. Fourth, oxidative stress is mitigated through the intake of antioxidants found in a diet rich in fruits and vegetables, which helps prevent the initiation and progression of cancer [42, 43]. Lastly, the improvement of gut microbiota through a balanced diet can significantly impact systemic inflammation and, consequently, cancer risk [44, 45]. These combined actions emphasize the importance of a comprehensive approach to health, highlighting how interconnected lifestyle factors contribute to both cardiovascular and cancer prevention.

The primary strength of this research is its introduction of a new perspective on the potential association between LE8 and subsequent cancer risk. The study also thoroughly accounted for potential confounders, including lifestyle factors and histories of cancer‐related diseases. Several limitations should be considered when interpreting our findings. First, the Kailuan study lacks comprehensive data on certain cancer‐related causal factors, such as hepatitis C virus infection for liver cancer, *Helicobacter pylori* for stomach cancer, and dietary patterns including whole grains, vegetables, and fiber intake for colorectal cancer. This restricts our ability to fully adjust for potential confounders. Second, although dietary data were collected, they were incomplete; surrogate indicators such as salt intake, tea consumption, and high‐fat diet frequency were used, which may not fully reflect the dietary components outlined in the AHA's LE8 guidelines. Moreover, some LE8 components were not calculated strictly according to standard definitions, potentially limiting comparability with other studies. Third, LE8 scores were assessed only at baseline, and changes in cardiovascular health status over time were not captured. Although participants underwent biennial health assessments, repeated LE8 measurements were not available for all individuals, and incorporating time‐varying exposures would have substantially reduced the sample size, thereby limiting statistical power. As a result, the baseline LE8 score may not fully reflect long‐term behavioral and biological changes that influence cancer risk. Future studies leveraging longitudinal LE8 data as it becomes increasingly available in this cohort could further elucidate the impact of dynamic changes in cardiovascular health on cancer outcomes. Fourth, the cohort was derived entirely from the Kailuan community—an industrial workforce with predominantly male, middle‐aged participants. Although all models were adjusted for sex and the Kailuan Group has diversified beyond coal mining into sectors like healthcare and education, a lack of occupational stratification and potential residual exposure to industrial carcinogens (e.g., coal dust) remain sources of unmeasured confounding. However, only a small proportion of participants were underground miners, and most were employed in surface or non‐mining roles. Lastly, while the Kailuan cohort may appear occupationally specific, it reflects a relatively high socioeconomic level in comparison to national averages at the time (https://data.stats.gov.cn/easyquery.htm?cn=C01), given its location in the economically developed city of Tangshan. Nonetheless, generalizability to the broader Chinese population should be made with caution.

## 5 Conclusions

This study demonstrates that the LE8 framework can significantly reduce the risk of various cancer types. Higher LE8 scores correlate with lower incidences of cancers such as lung, breast, colorectal, kidney, and endometrial. These results underline the importance of incorporating LE8 into public health and clinical cancer prevention strategies, emphasizing holistic lifestyle modifications to decrease cancer risk. The research advocates for the broader use of LE8, supporting a preventive approach in healthcare that promises significant public health benefits.

## Author Contributions

**Li Deng:** methodology, software, and writing – original draft preparation. **Xiao‐Meng Hao:** writing – reviewing and editing. **Zhe Shu:** supervision and validation. **Tong Liu:** methodology. **Hong‐Tao Wang:** data curation, software, and investigation. **Qing‐Song Zhang:** conceptualization and supervision. **Han‐Ping Shi:** conceptualization and supervision, validation, resources. The corresponding author attests that all listed authors meet authorship criteria and that no others meeting the criteria have been omitted. All the work reported in the paper has been performed by the authors unless clearly specified in the text.

## Funding

This work was supported by the National Key Research and Development Program (2022YFC2009600 and 2022YFC2009601); Laboratory for Clinical Medicine, Capital Medical University (2023‐SYJCLC01 and 2023‐SYJCLC02); National Multidisciplinary Cooperative Diagnosis and Treatment Capacity Project for Major Diseases: Comprehensive Treatment and Management of Critically Ill Elderly Inpatients (No. 2019.YLFW) to Dr. Hanping Shi. The funders had no role in considering the study design or in the collection, analysis, interpretation of data, writing of the report, or decision to submit the article for publication.

## Disclosure

Transparency statement: The manuscript's guarantor affirms that this manuscript is an honest, accurate, and transparent account of the study being reported, that no important aspects of the study have been omitted, and that any discrepancies from the study as planned (and, if relevant, registered) have been explained.

## Ethics Statement

This study obtained ethical approval from the ethics committees of Kailuan General Hospital and Beijing Shijitan Hospital, and it adhered to the principles outlined in the Declaration of Helsinki. Written informed consent was obtained from participants or their legal representatives. The Kailuan Study was retrospectively registered in the Chinese Clinical Trial Register on August 24, 2011 (ChiCTR–TNRC–11001489; http://www.chictr.org.cn/showprojen.aspx?proj=8050).

## Conflicts of Interest

The authors declare no conflicts of interest.

## Supporting information


**Data S1:** cam471518‐sup‐0001‐Supinfo.docx.

## Data Availability

Data will be made available upon reasonable request.
