# Supplementary material for: Life's Essential 8 and the Risk of Overall and Specific‐Site Cancer Types: A Large‐Scale Prospective Cohort Study
Source: Cancer Med. 2026 Jan 19;15(1):e71518. doi: 10.1002/cam4.71518 (PMC12815607; doi:10.1002/cam4.71518)

**Supplementary materials**

**Supplementary table 1. Definition and scoring approach for LE8**

| **Domain** | **Metric** | **Method of measurement** | **Quantification of LE8** | | |
| --- | --- | --- | --- | --- | --- |
| **Health** | **Diet** | Measurement: | Scoring in the Kailuan cohort: | | |
| **behaviors** | **Health** |  | Salt scoring: | | |
|  |  | Self-reported intake of salt, fatty | <6g /day (33.3 points); | | |
|  |  | foods, and tea in the Kailuan cohort: | 6-12 g/day (16.7 points); | | |
|  |  | Examples of measurement: “What | >12 g/day (0 points) | | |
|  |  | flavor do you prefer?”” How often | Fatty food scoring: | | |
|  |  | do you eat fatty foods?” | <1 time/week (33.3 points); | | |
|  |  | “How often do you drink tea?” | 1-3 times/week (16.7 points); | | |
|  |  |  | >3 times/week (0 points) | | |
|  |  |  | Tea scoring: | | |
|  |  |  | > four times/week (33.3 points); | | |
|  |  |  | 1-3 times/week (25 points); | | |
|  |  |  | 1-3 times/month (16.7points); | | |
|  |  |  | <1 time/month (8.33 points); | | |
|  |  |  | Never (0 points) | | |
|  |  |  | The final diet score is the sum of its component | | |
|  |  |  | scores. | | |
|  | **Physical** | Measurement: Self-reported times of | Metric: | | |
|  | **activity** | physical activity per week. | Minutes of physical activity per week in the Kailuan | | |
|  |  | Example tools for measurement: | cohort. | | |
|  |  | “How many times did you usually | Scoring in the Kailuan cohort: | | |
|  |  | spend on physical activity (note: It | Points Minutes | | |
|  |  | took at least 20 minutes each time)?” | 100 ≥80 | | |
|  |  | in the Kailun cohort. | 50 20-60 | | |
|  |  |  | 0 <20 | | |
|  | **Nicotine** | Measurement: Self-reported use of | Metric: Smoking status | | |
|  | **exposure** | cigarettes |  | | |
|  |  | The age of starting and quitting |  | | |
|  |  | Example tools for measurement: Do | Scoring in the Kailuan cohort: | | |
|  |  | you now smoke cigarettes? (Never | Points Status | | |
|  |  | smoker, former smoker, some days, | 100 | Never smoker |  |
|  |  | every day) | 50 | Former smokers quit ≥l year |  |
|  |  | The age of starting and quitting | 25 | Current smokers, < l cigarette/day |  |
|  |  | smoking | 0 | Current smoker, ≥l cigarette/day |  |
|  | **Sleep** | Measurement: Self-reported average | Metric: Average hours of sleep per night | |  |
|  | **health** | hours of sleep per night | Scoring: |  |  |
|  |  | Example tools for measurement: “On | Points | Hour |  |
|  |  | average, how many hours of sleep do | 100 | 7-< 9h |  |
|  |  | you get per night in the last year?” | 90 | 9-< 10h |  |
|  |  |  | 70 | 6-< 7h |  |
|  |  |  | 40 | 5-<6 or ≥10h |  |
|  |  |  | 20 | 4-<5 h |  |
|  |  |  | 0 | < 4h |  |
| **Health** | **Body mass** | Measurement: Body weight (kg) |  | Metric: Body mass index (kg/m²) |  |
| **factors** | **index** | divided by height squared (m²) | Scoring: |  |  |
|  |  | Example tools for measurement: | Points | Level |  |
|  |  | Objective measurement of height and | 100 | <23 |  |
|  |  | weight | 75 | 23.0-24.9 |  |
|  |  |  | 50 | 25.0-29.9 |  |
|  |  |  | 25 | 30.0-34.9 |  |
|  |  |  | 0 | ≥35.0 |  |
|  | **Blood** | Measurement: Plasma total and HDL | Metric: Non-HDL cholesterol(mmol/L) | |  |
|  | **lipids** | cholesterol with the calculation of |  |  |  |
|  |  | non-HDL cholesterol. | Scoring: |  |  |
|  |  |  | Points | Level |  |
|  |  | Example tools for measurement: | 100 | <3.36 |  |
|  |  | Fasting blood sample. | 60 | 3.36-4.13 |  |
|  |  | Non-HDL-cholesterol unit | 40 | 4.14-4.90 |  |
|  |  | conversion: | 20 | 4.91-5.68 |  |
|  |  | 1mmol/L= 38.67mg/L | 0 | ≥5.69 |  |
|  |  | 1mg/L=0.02586mmol/L | If the drug-treated level subtracts 20 points | |  |
|  | **Blood** | Measurement: Fasting blood |  | Metric: FBG (mmol/L) |  |
|  | **glucose** | glucose (FBG) | Scoring: |  |  |
|  |  | Example tools for measurement: | Points | Level |  |
|  |  | Fasting blood glucose sample. | 100 | No history of diabetes with FBG<5.6 |  |
|  |  | HBAlC to FBG(mg/L) to | 60 | No diabetes with FBG 5.6-6.9 |  |
|  |  | conversion: | 40 | Diabetes with FBG<8.6 |  |
|  |  | 28.7*A1C-46.7=FBG | 30 | Diabetes with FBG 8.6-10.1 |  |
|  |  | FBG unit conversion: | 20 | Diabetes with FBG 10.2-11.6 |  |
|  |  | 1mg=0.056mmol/L | 10 | Diabetes with FBG 11.7-13.2 |  |
|  |  | 1mmol/L=18.02mg/dL | 0 | Diabetes with FBG≥13.3 |  |
|  | **Blood** | Measurement: Appropriately | Metric: Systolic and diastolic blood pressure(mmHg) | |  |
|  | **pressure** | measured systolic and diastolic blood |  |  |  |
|  |  | pressure | Scoring: |  |  |
|  |  |  | Points | Level |  |
|  |  | Example tools for measurement: | 100 | <120/<80 |  |
|  |  | Corrected Mercury sphygmomanometer | 75 | 120-129/<80 |  |
|  |  |  | 50 | 130-139 or 80-89 |  |
|  |  |  | 25 | 140-159 or 90-99 |  |
|  |  |  | 0 | ≥160 or≥100 |  |
|  |  |  |  | Subtract 20 points if the treated level |  |

**Note: Modified from American Heart Association's New “Life’s Essential 8TM” Metrics.**

**Supplementary table 2. Cancer Diagnosis Classification (ICD-10)**

| **Cancer Type** | **ICD-10 Codes** |
| --- | --- |
| Head and Neck Cancer | C00-C14, C30-C32, C71, C73 |
| Esophageal Cancer | C15 |
| Stomach Cancer | C16 |
| Small Intestine Cancer | C17 |
| Colorectal Cancer | C18-C21 |
| Liver Cancer | C22 |
| Gallbladder or Extrahepatic Bile Duct Cancer | C23-C24 |
| Pancreatic Cancer | C25 |
| Lung Cancer | C34 |
| Bone and Soft Tissue Cancer | C40-C41, C49 |
| Skin Cancer | C43-C44 |
| Breast Cancer | C50 |
| Cervical Cancer | C53 |
| Uterine Cancer | C54-C55 |
| Ovarian Cancer | C56 |
| Prostate Cancer | C61 |
| Kidney Cancer | C64-C65 |
| Bladder Cancer | C67 |
| Lymphoma | C81-C89 |
| Leukemia and Multiple Myeloma | C90-C96 |

**Supplementary table 3. Sensitivity analyses of adjusted HRs and 95% CIs for risk of specific-site cancer across the clinical cutoffs of life’s essential 8 scores in Kailuan Study**

|  | **Each 10-points increment** | **LE8 scores by cutoff** | | | **P for trend** |
| --- | --- | --- | --- | --- | --- |
|  |  | **Poor (<50)** | **Intermediate (50–79)** | **Ideal (≥80)** |  |
| **Exclude participants with cancer diagnosed within 1st year of follow-up (n=93,940)** | | | | | |
| **Overall cancer** | **0.94 (0.91, 0.96)** | Ref. | **0.88 (0.80, 0.97)** | **0.74 (0.64, 0.85)** | **<0.001** |
| **Lung cancer** | **0.90 (0.85, 0.95)** | Ref. | **0.80 (0.68, 0.95)** | **0.69 (0.53, 0.91)** | **0.013** |
| **Breast cancer ^a^** | **0.87 (0.76, 0.99)** | Ref. | **0.40 (0.25, 0.65)** | **0.37 (0.21, 0.65)** | **0.001** |
| **Kidney cancer** | **0.83 (0.73, 0.95)** | Ref. | 0.83 (0.54, 1.28) | **0.45 (0.22, 0.97)** | 0.217 |
| **Colorectal cancer** | **0.92 (0.85, 0.99)** | Ref. | 0.85 (0.67, 1.07) | **0.46 (0.30, 0.72)** | **0.003** |
| **Endometrial cancer ^a^** | **0.77 (0.61, 0.96)** | NA | NA | NA | NA |

**Note: Adjusted models include age, gender, CRP, drinking status, marital status, family income, and sedentary lifestyles.**

**a: Conducted only in women.**

**Results presented with bold valued were statistically significant with all p value < 0.05.**

**Supplementary figure 1. The flowchart of the study population in Kailuan Study**


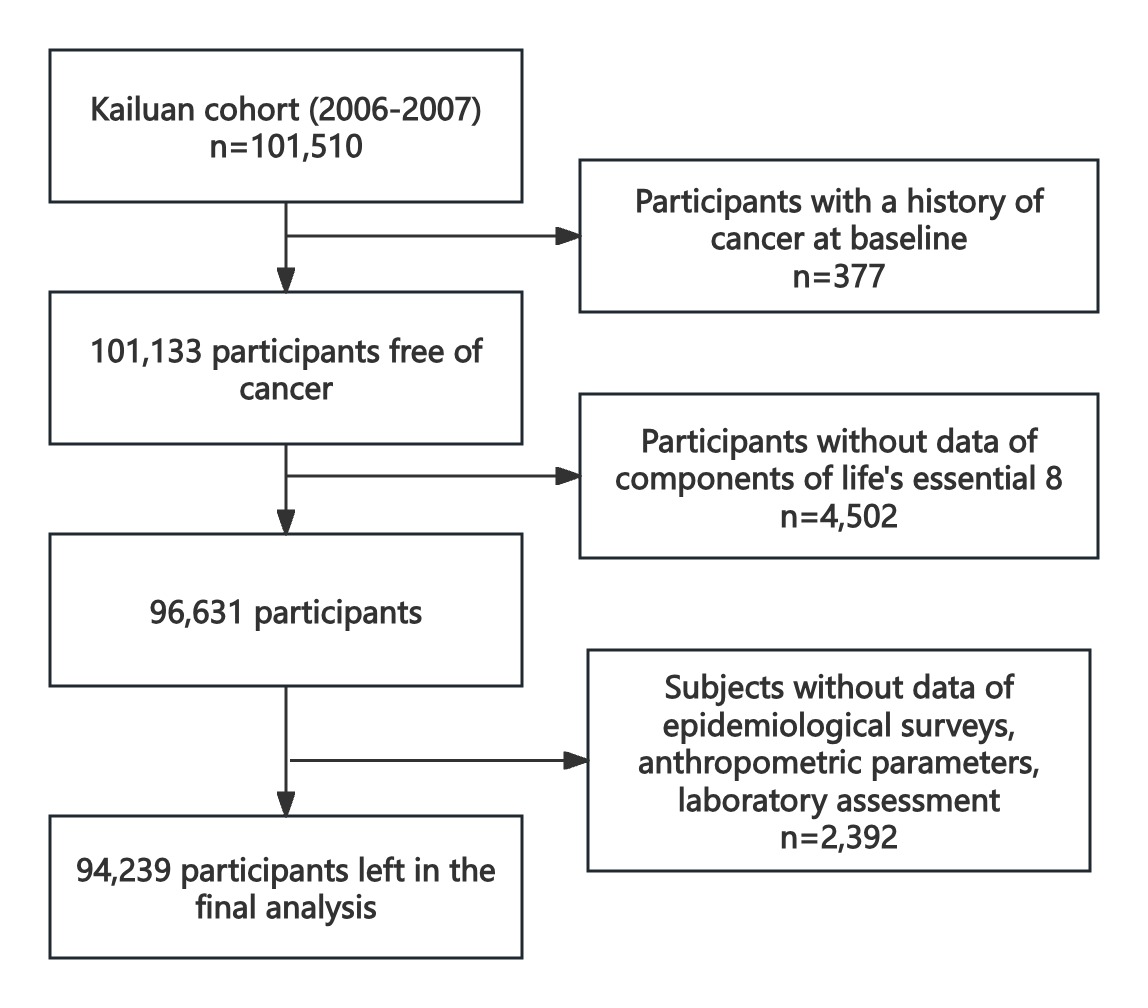

Supplement: Supplementary file 1 — Data S1: cam471518‐sup‐0001‐Supinfo.docx. [file CAM4-15-e71518-s001.docx]
